# Utilization of Construction and Demolition Mix Waste in the Fired Brick Production: The Impact on Mechanical Properties

**DOI:** 10.3390/ma16010262

**Published:** 2022-12-27

**Authors:** Mandefrot Dubale, Milica Vidak Vasić, Gaurav Goel, Ajay Kalamdhad, Laishram Boeing Singh

**Affiliations:** 1 Department of Civil Engineering, Indian Institute of Technology Guwahati (IITG), Guwahati 781039, India; 2 Institute for Testing of Materials IMS, Bulevar Vojvode Mišića 43, 11000 Belgrade, Serbia; 3 School of Energy and Environment, Thapar Institute of Engineering and Technology, Patiala 147004, India

**Keywords:** mixed C&D waste, clay brick, fired brick quality, laterite soil, alluvial soil, firing temperature

## Abstract

The European Green Deal, which emphasizes zero-waste economies, and waste recycling in construction and building materials, has arisen due to significant worldwide needs for solid waste recovery and usage. This ambitious study focuses on recycling mixed construction and demolition (C&D) waste into burnt bricks and investigating the influence of firing temperature. While pursuing its objectives, this is dependent on raw material characterization and burnt-brick product quality assessment. The recycling of mixed C&D waste is explored by mixing the waste into two soil types (alluvial and laterite) in ratios ranging from 5% to 45% at three firing temperatures (700 °C, 850 °C and 900 °C). The utilization of mixed C&D waste in amounts of 10% at 700 °C and 25% at 850 °C and 900 °C fulfilled the Indian standard. Although a fire at 700 °C results in less optimal waste utilization, it is beneficial and recommended for reducing the carbon footprint and energy use. Additional mineralogical and microstructural analyzes are performed on the optimal fired samples. The study’s findings are promising for sustainable resource usage, reducing carbon footprint, and reducing waste disposal volume. This research is a big step toward the Sustainable Development Goals of the United Nations and a circular economy.

## 1. Introduction

Global emphasis is on the recycling of waste materials to achieve zero waste. Population growth, lifestyle changes, technological advancements, and other factors of modernization all have a significant impact on waste generation. The development in urbanization, as well as city reconstruction, is contributing to the increase in construction and demolition (C&D) waste. It is estimated that approximately 30% to 40% of solid waste generated comes from C&D waste [1,2,3,4]. Waste from C&D operations has received a great deal of attention, becoming the focal point of environmental effect, and about 85% of it is not recycled; 35% is utilized as landfill. [5]. The key features of C&D waste include inert and non-inert behavior, dust particle discharge into the air, huge volume, unsorted and complicated components, and a substantial percentage of nonbiodegradable material that needs future study [6,7]. C&D waste is typically disposed of in unplanned and unauthorized locations, while demand for natural resources utilized in construction continues to grow [8,9]. In 2012, the global generation of C&D waste was predicted to be approximately 3 billion tons. China, India, and the US are the top three nations in terms of C&D waste production [2]. Waste production in Europe is predicted to be 0.175 billion tons per year, whereas waste production in developing nations is estimated to be 1.825 to 2.825 billion tons per year [10]. As a consequence, appropriate waste disposal, C&D waste management, and waste recycling are necessary.

India has 18% of the world’s population and is one of the world’s fastest-growing economies. The construction sector is the country’s growth engine, accounting for the second-largest economic activity. It accounts for around 10% of the country’s total gross domestic product (GDP). It is estimated that around 63 million people, including both urban and rural populations, live in housing that is inadequate for their requirements. As a consequence of this circumstance, a substantial quantity of waste is generated around the country [11,12]. The researchers estimate that 112 to 700 million tons of C&D waste are created each year, but this figure is not currently effectively recorded [7]. The rates of C&D waste disposal in Delhi, Mumbai, and Kolkata are 5000, 3000, and 2000 tons per year, respectively. These three cities are at the top of the list of metro cities in terms of C&D waste generation. In 2016, India developed a waste management policy that includes C&D waste; moreover, the country has already begun trying to encourage its stakeholders to recycle solid waste products. Nonetheless, according to the report, India would only use 1% of all C&D waste in the country by 2020 [7].

Recycling C&D waste and its use in construction materials are becoming an increasingly important area of study in the modern day. Several studies have been carried out to investigate the viability of using various wastes in construction and building materials. Waste foundry sand (WFS) is considered to be one of the wastes that are recycled into building materials. It is a by-product of the metal casting industry and is high-quality silica sand that is black in color. The usage of WFS introduces poor strength in concrete and asphalt concrete during the partial substitution of natural sand. In addition, it was discovered that the compressive strength and the modulus of elasticity were both satisfactory when fine aggregate was being replaced [13,14,15]. Coal bottom ash (CBA) and fly ash are two additional types of waste that are utilized as a partial substitute for sand in the manufacture of concrete. Because of their appearance, particle size, and silico-aluminous nature, both are tempting in the recycling process of concrete as a partial substitute for sand. They are by-products of industrial waste from coal-fired thermal power plants. According to research conducted on the replacement of fine aggregate on concrete production of CBA, addition at 50% and 100% increased fine particle content, water consumption, internal friction, setting time, and compressive strength. Flexural strength, on the other hand, remained constant, whereas split tensile strength decreased [13,16,17]. Fly ash is also being investigated for use in a variety of applications, including cement and concrete production [17], structural infill material [18], pavement [19,20], asphalt mixture [21], and fired bricks [22].

There is the possibility for good change in the world if C&D waste is utilized as a partial substitute in construction and building materials. Despite this, several studies exploring the feasibility of utilizing C&D waste in construction materials have been conducted (Table 1). One of the many successful recycling applications is the use of C&D waste as aggregate in the production of concrete. According to the researchers’ findings, adding 10 mm aggregate size to concrete leads to more water absorption, reduced specific gravity, lower bulk density, and increased compressive strength. To improve the way the components of C&D waste are linked together, the waste’s characteristics must have a high water absorption rate [23,24]. The use of 3% of recycled concrete aggregate and 1% of crushed brick as a material in either a subbase or a pavement produced favorable results in terms of the deformation behavior [25]. Clay brick that has been demolished may be used instead of geo-polymer binder in the construction of pavement [26]. The usage of this waste for the production of fired brick, on the other hand, is not nearly as high when compared to its integration into concrete. The use of construction waste in the manufacturing of bricks at 10% to 20% resulted in improved extrusion, a reduction in linear firing shrinkage, a drop in working moisture, and an improvement in mechanical strength [27]. It is believed that incorporating 30% to 40% of the demolished ceramic roof and wall tile waste into the production of fired bricks is one of the most beneficial and sustainable ways to safeguard natural resources [28]. Insufficient exploitation of C&D waste in the production of burnt bricks is one of the gaps and unplanned areas are commonly used for dumping purposes. At the same time, the widespread utilization of waste in construction and building materials demonstrates a positive impact on sustainable development, reduces the amount of waste that is disposed of, and presents a satisfying solution for reducing the negative impact that humans have on the environment [29].

The data presented in Table 1 [29,30,31,32,33,34] relate to comparable work that had been carried out in earlier research, together with the optimal requirements. Compressive strength, water absorption, firing linear shrinkage, and bulk density are the characteristics of burned bricks that are most often measured and most frequently examined. All of the research that has been done on the inclusion of C&D waste in burned brick manufacturing have shown positive outcomes. This research study is, to the best of our knowledge, the only one of its kind to undertake mixed C&D waste testing in two distinct kinds of soil and at three firing temperatures.

For this study, a partial replacement of the natural clay soil that is normally utilized in the manufacturing of fired brick was substituted by mixed C&D waste. The originality of the study consists of the use of two different soil types that are typically utilized in the Indian subcontinent for the production of bricks, as well as the examination of many parameters that influence the quality of bricks. The objective of the study was to (1) use the mixed C&D waste; (2) analyze the influence of firing temperature to discover the optimal firing temperature, and (3) limit the usage of natural resources while simultaneously increasing the adoption of environmentally friendly solutions. This effort will be beneficial in reaching sustainable development objectives since waste management is necessary for healthy living on land, which is one of the Sustainable Development Goals (SDG 15) [35].

## 2. Materials and Methods

When making fired bricks, mixed construction and demolition debris is often utilized as a partial replacement for raw clay. In the brick manufacturing sector of India, laterite and alluvial soil are regarded as the two natural resources that are believed to be of the utmost importance. They are necessary to the brick manufacturing industry in India and come highly recommended as a result of the high percentage of clay minerals and fine particles that they contain. In addition, they have low levels of nitrogen, phosphorus, and organic matter in their composition, which also contributes to their superiority. Alluvial soil (AS) was collected from the nearby Brahmaputra River and its transportation, loading, and unloading are done by daily laborers. At the same time, laterite soil (LS) was collected from inside the campus. The C&D mixture was obtained and analyzed by the researchers using materials such as demolished concrete, marble, fired clay bricks and blocks, ceramic tiles, asphalt, and roofing tiles. As a consequence of the renovation work that was being performed, mixed C&D waste was collected from the backyards of various hostels located on the campus as well as from the villages that are located near the university. After collecting, there is no separation performed. The mixed C&D waste was milled using a ball-rolling grinder and sieved to a fraction of less than 0.6 mm and it was the same for both soils. Preparing the raw materials is followed by raw material characterization and brick product quality characterization. The chemical and mineralogical compositions of both soils are shown elsewhere [36].

### 2.1. Raw Material Characterization

The chemical composition of the mixed C&D waste was recorded by using X-ray fluorescence (XRF) spectroscopy (PANalytical AXIOS Sequential XRF Spectrometer). The analysis was carried out at the instrumental section of the University of Guwahati (sophisticated analytical instrument facility (SAIF), Guwahati, India).

A mineralogical characterization test for the mixed C&D waste was performed by X-ray diffraction (XRD) at the Central Instrumental Facility (CIF) at the Indian Institute of Technology Guwahati (IITG). The 9KW Powder X-ray Powder Diffraction machine (Rigaku Technologies, Tokyo, Japan, Model: Smartlab). The X-ray wavelength (1.54184 Å) is produced by Cu K-ά.

Determining the toxic metals leaching from mixed C&D waste is conducted by toxicity characteristics leaching procedure (TCLP) test [37]. The Environmental Protection Agency (EPA, Washington, DC, USA) used method 1311 guidelines for the determination of toxic elements such as As, Cd, Cr, Cu, Fe, Ni, Mn, Pb, and Zn. Atomic absorption spectrometry was used for the measurements (Atomic Absorption Spectrometer Thermo Scientific, Waltham, MA, USA, iCE 3000).

Identification of the functional groups in the raw material is performed by Fourier-transform infrared (FTIR) spectroscopy (IRAffinity-1; M/s Shimadzu, Japan).

Differential thermal and thermogravimetric analysis (DTA/TGA, Netzsch STA 449F3A00 Instrument from 30 °C to 1000 °C) was used to investigate the weight change and thermal stability caused by a firing temperature heating rate of 10 °C/min in a static nitrogen atmosphere. This was done using the instrument from 30 °C up to 1000 °C.

Microstructural morphology of mixed C&D waste was investigated using Zeiss Sigma 300 field-emission scanning electron microscopy (FESEM) at CIF, with magnification ranging from 10× to 300,000×.

**Figure 1:**
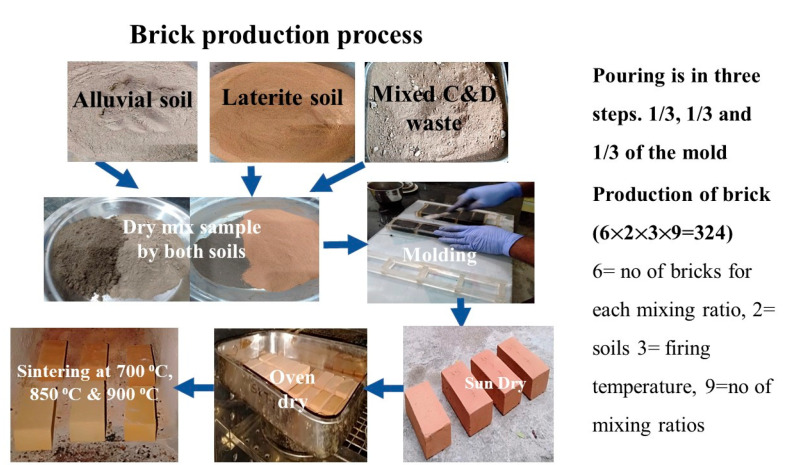
Fired brick production process.

The amount of mixed C&D waste that could be incorporated into two different soils can vary from about 5% to 45%. The percentage of soils that are being used in the production of fired bricks drops from 95% to 55%. To obtain a material that is uniform throughout, it is necessary to perform an adequate dry mixing action during the process of making fired bricks. To get the desired consistency, which is achieved by adding 20% to 25% more water, the waste and the soil were mixed with water before being added. To ensure effective manual pressing, the wet mixture was poured into the cuboid-shaped laboratory-scale molding in three different layers that were evenly spaced apart. The wet bricks were exposed to the sun for one day, dried in an oven at a temperature of 105 ± 5 °C the following day, and then burned in an electrically operated muffle furnace for five hours at three temperatures: 700 °C, 850 °C, and 900 °C. These temperatures are representative of the typical firing temperatures used in commercial kilns in India. Figure 1 provides an overview of the manufacturing process. Brick characteristics were measured on all of the pieces, and six samples of bricks were produced for each different mixing percentage of alluvial and laterite soil.

### 2.2. Properties of Bricks

A total of 324 samples was produced for the study: two types of soils, three final firing temperatures, 9 shares of the waste, and 6 samples for testing.

Using a digital Vernier caliper, the dimensional change of all produced bricks was tracked to calculate the firing shrinkage that occurred during the sintering process. Before and after the brick sample was fired, the dimensions were measured both times. Loss on ignition was carried out to determine the weight loss, which was primarily the result of the removal of water, organic matter and carbonates from the sample. It is calculated as the percentage difference between the weight loss before and after firing the brick sample [38,39,40].

The purpose of the bulk density measurement is to determine how much the volume has changed concerning the ratio of the wet mass before and after the bricks have been sun-dried and fired in the muffle furnace. The quantity of water that is absorbed by the brick sample is referred to as its water absorption, and it can be determined by immersing it for 24 h in water at room temperature. After allowing the bricks to soak for 24 h, they were removed from the water, wiped down with a damp cloth, and then immediately weighed. A universal testing machine (UTM, 250 kN) was used to determine the compressive strength of specimens of fired brick. The load was applied to the samples continuously and uniformly until they broke, and the machine is automatically adjustable to ensure that it uniformly transmits the load. Tests were performed on five samples representing each mixing ratio, and the average results were reported. To investigate apparent porosity, the ASTM C20 [41] standard was utilized, while the Indian standard IS: 3495 [42] was utilized to investigate the sample’s efflorescence. The results on the compressive strength and water absorption are presented as the average of 3 samples.

A mineralogical determination was performed on the optimal fired samples by using the XRD instrument by Rigaku Technologies, Japan.

Micromorphology of those samples was investigated by Zeis Sigma 300 FESEM, with magnification ranging from 10× to 300,000×. The samples to be tested were primarily covered with a layer of gold using a coating spray device for enhanced reflection.

## 3. Results

### 3.1. Raw Materials

#### 3.1.1. Mineralogical Composition of the C&D Waste

**Figure 2:**
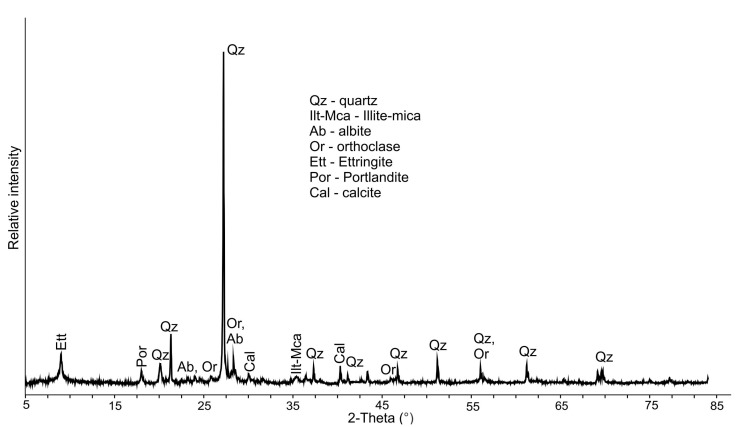
XRD result of unfired demolished mixed C&D waste.

The mineralogical composition of the used soils is presented elsewhere [43]. The main difference is that laterite soil is kaolinitic with some montmorillonite, while alluvial soil is kaolinitic–illitic. The XRD pattern produced from mixed C&D waste is shown in Figure 2. It does this by using a highly sensitive dual imaging plate technology, which not only increases the accuracy of the readings but also reduces the amount of time that is required to gather the data. The XRD graph reveals that the main peak related to quartz is very prominent, and it also demonstrates that mixed C&D waste includes quartz as the major crystalline phase [44]. In addition, some illite–mica and feldspars (albite and orthoclase) are detected. The mineral phases originating from concrete are found to be ettringite, portlandite and calcite. However, ceramic-based products made up the majority of the waste that was examined.

#### 3.1.2. Chemical Composition of the C&D Waste

The findings of the XRF analysis of laterite soil, alluvial soil, and mixed C&D waste are shown in Table 2 as interims of main oxides. In all raw materials, the primary oxide elemental components in XRF analysis are SiO_2_ and Al_2_O_3_, as expected. Given that concrete contains a large proportion of CaO, these results indicate that the share of this material in the mixed C&D waste was relatively low. The presence of fluxing agents was the highest in laterite soil and the lowest in mixed C&D waste. Bricks may be made with any other raw material as long as it has a high proportion of silicon dioxide (between 50% and 60%) and aluminum oxide (between 20% and 30%) as their main chemical components. The mixed C&D waste in these mixtures acts as a filler since it lowers the content of clay minerals in the matrix, lowers plasticity, and decreases drying shrinkage [39].

#### 3.1.3. Fourier-Transform Infrared Spectrometry (FTIR)

**Figure 3:**
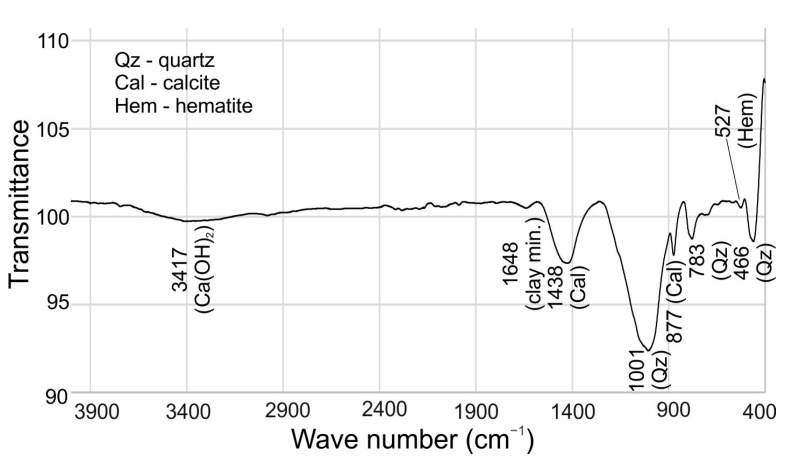
FTIR result of the mixed C&D waste.

FTIR is a spectrum graph that is produced by applying four distinct modes of molecular vibration (bending, rocking, twisting, and scissoring) [45]. The XRD and XRF results were constrained by the mineralogy observed by FTIR. The main molecular bands identified with FTIR are shown in Figure 3; and they are detected at approximately 3417 cm^−1^, 1438 cm^−1^, 1001 cm^−1^, 877 cm^−1^, 783 cm^−1^, and 466 cm^−1^. The strong band situated at 1001 cm^−1^ represents the asymmetric stretching internal vibrations of silica and/or alumina bonded to the oxygen atom (Si-O-Si and Si-O-Al) and is considered mostly related to the content of quartz, but also feldspars and clay minerals [46,47]. Other smaller bands, also characteristic of quartz presence, are found at 466 and 783 cm^−1^ [46,48]. The detected bands corresponding to C-O vibration in CaCO_3_ are found at 877 and 1438 cm^−1^. Additionally, the broad and very weak band from -OH vibration in Ca(OH)_2_ was seen at about 3417 cm^−1^ [49,50]. A small band found at 1648 cm^−1^ was assigned to a small content of clay minerals in the material. Also, a weakly prominent peak at 527 cm^−1^ showed the presence of hematite [48]. The FTIR analysis and the missing characteristic bands at about 3600 cm^−1^ and 1600 cm^−1^ show that there is a very low amount of clay minerals in the studied waste [48].

#### 3.1.4. Thermal Behavior

**Figure 4:**
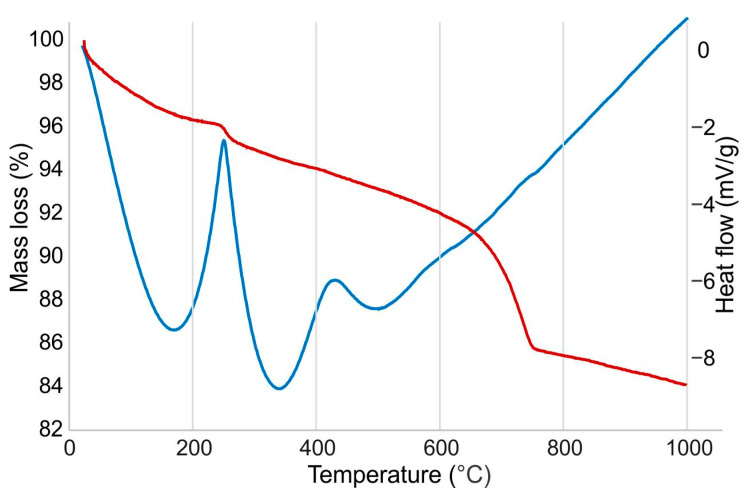
DTA/TGA curves of mixed C&D waste.

Figure 4 depicts the thermal analysis (DTA/TGA) of the mixed C&D waste at a temperature ranging from 30 to 1000 °C. The study is done in a dynamic manner, which means that the temperature rises continuously at a constant heating rate [51].

The weight loss is approximately 4% when the temperature is raised from 30 °C to 200 °C due to the removal of free water and interlayer OH-groups, and trapped carbon dioxide. The burning of organic matter and impurities resulted in a ~2% weight loss from 200 to 400 °C, while the total weight loss is about 16%. The main exothermal reaction appeared at about 255 °C resulting from organic matter decomposition while being interrupted by an endothermic reaction at about 343 °C, which is due to the decomposition of ettringite [52]. Another endothermic effect noticed at 501 °C might be caused by the decomposition of portlandite. The characteristic endothermic peaks of the clay mineral decomposition at about 530–540 °C [46] were not seen in the DTA curve. The structural conversion of quartz is not seen in the DTA graph since it is overlapped with more intensive reactions of dehydration. The same is with the decarbonization of carbonates, which is detected only in the corresponding TGA curve at 762 °C.

#### 3.1.5. Microstructure

**Figure 5:**
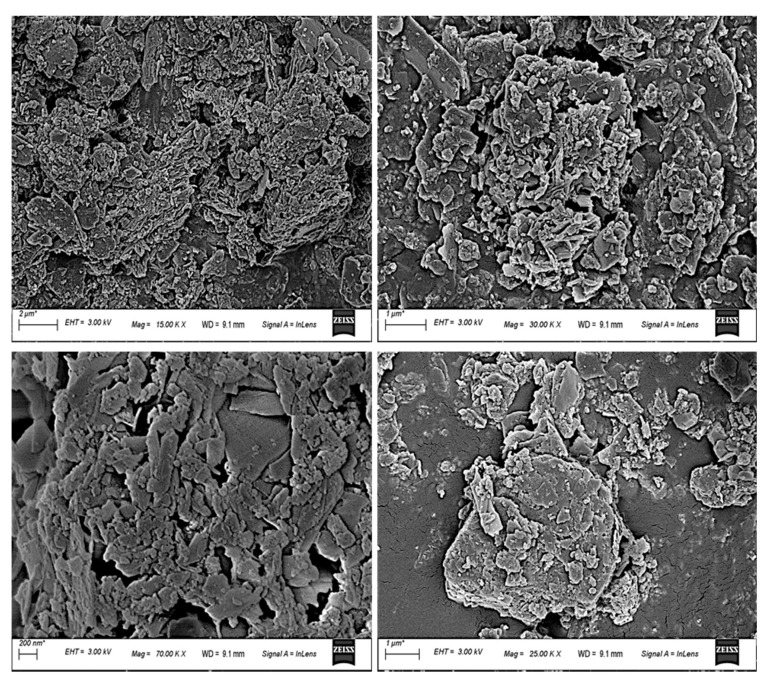
Microstructure morphology of parts of the mixed C&D waste at different magnifications.

Figure 5 displays the results of the examination of the microstructural morphology of mixed C&D waste. Micromorphology mostly consists of agglomerated material, which also exhibits some porosity and just a few occasional microscopic fissures. Agglomerates show a morphology made up of sub- and micron-sized particles. Also, some structures of ettringite that resemble whiskers are seen. A hard alumina–silicate phase is present in the material after it has been fused. Bricks that are created by adding the waste would be improved in their vitrification quality if an additional fluxing agent is included in the mixed construction and demolition debris [53].

#### 3.1.6. Toxicity Characteristics Leaching Procedure (TCLP)

The leachates that were created from the mixed construction and demolition waste exhibited levels that were much lower than the limitations stipulated by the Indian Hazardous Waste Management Rules (2016). As a consequence of this, the utilization of mixed C&D waste in the manufacturing of burnt brick is shown to be an ecologically responsible alternative. The results of the TCLP leaching tests are shown in Table 3.

### 3.2. Properties of Fired Bricks

**Figure 6:**
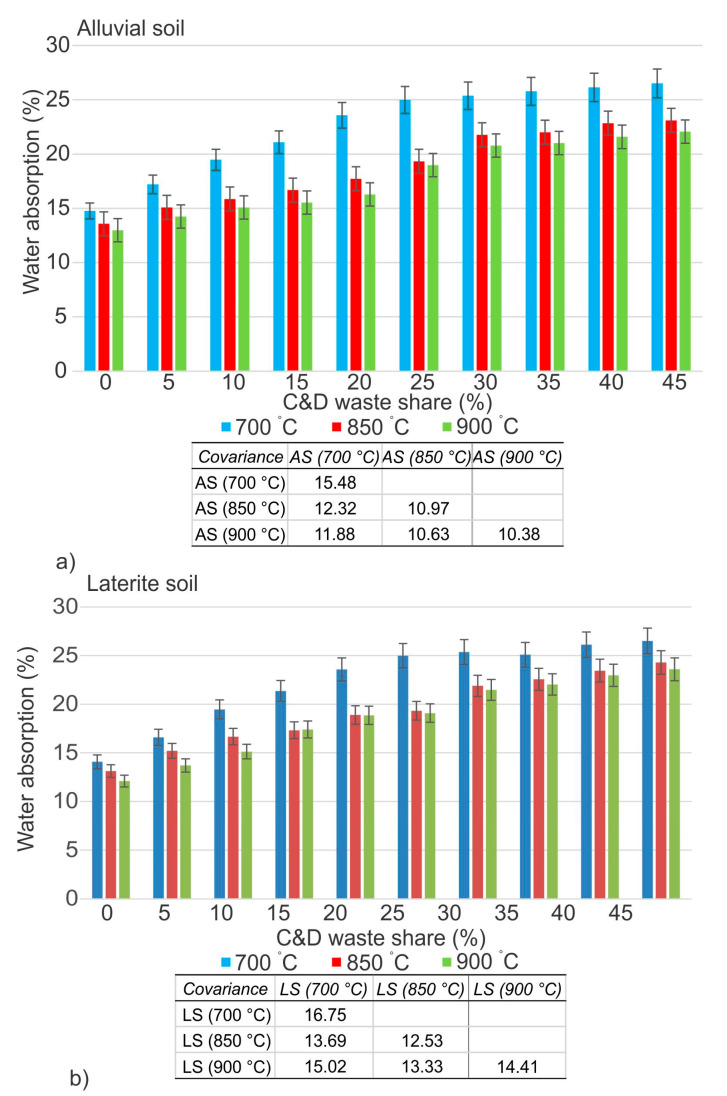
Water absorption of mixed C&D waste in alluvial (**a**) and laterite (**b**) soil.

A variety of factors are used to determine the mechanical and physical qualities of bricks. Compressive strength, water absorption, linear shrinkage, loss on igniting, and bulk density are some of the most important. The capacity of a material to withstand wear and tear may be impacted by characteristics such as water absorption and compressive strength. The higher quantity of mixed C&D waste is steadily increasing water absorption and also decreasing compressive strength. According to both Indian and ASTM standards [54,55], the maximum allowable water absorption is 20%. The findings are shown in Figure 6, and they demonstrate that incorporating 10% of mixed C&D waste into both soils after burning at 700 °C is acceptable and within the limitations. On the other hand, at temperatures of 850 °C and 900 °C, the inclusion of 25% mixed C&D waste is permitted on both soil types. The use of mixed C&D waste in soils showed that the addition of waste consistently increases water absorption at all three temperatures, as it induced porosity caused by somewhat increased contents of carbonates in the mixtures [28,56].

**Figure 7:**
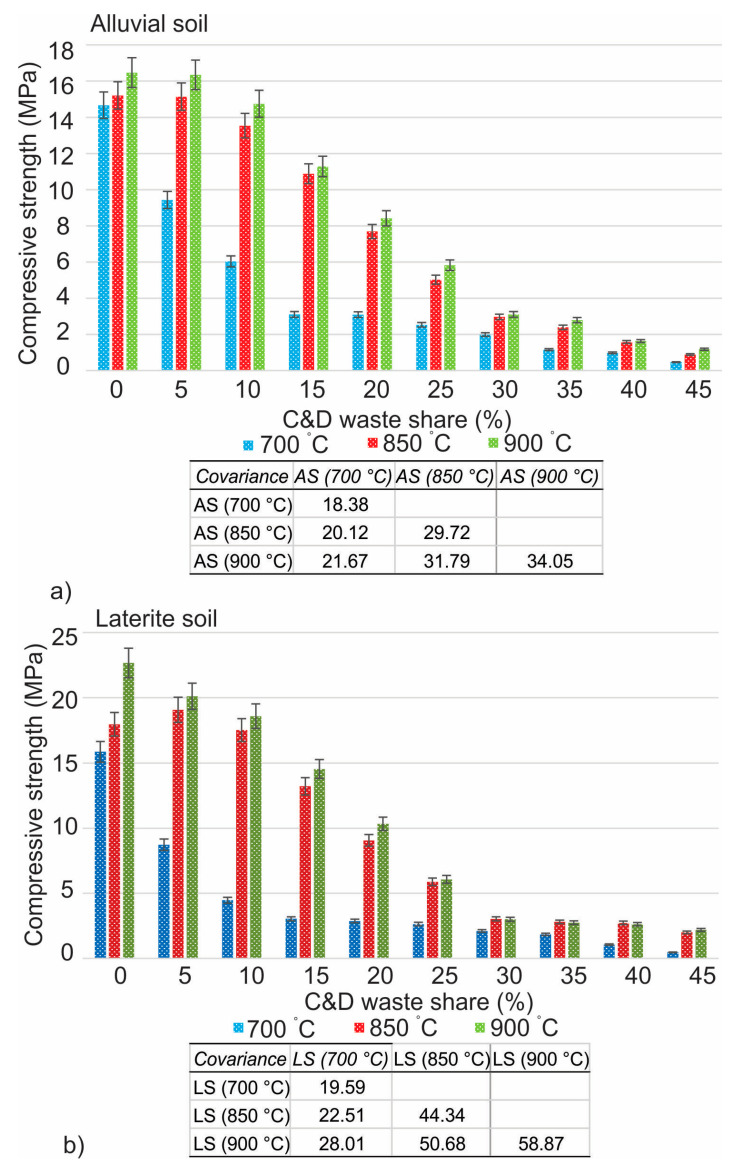
Compressive strength of mixed C&D waste in alluvial (**a**) and laterite (**b**) soils.

The compressive strength of all five specimens is displayed in Figure 7, which shows the average of all of the tested compressive strengths. According to the American standard (ASTM), the compressive strength of fired brick must be above 10.3 MPa, but according to the Indian standard (IS), it is to be a minimum of 3.5 MPa is required [54,55]. The weather in India is significantly different from that in North America, which is the reason for this difference. At a temperature of 700 °C, 10% had reached the limit, while at temperatures of 850 °C and 900 °C, 25% had satisfied the limit. At a temperature of 700 °C, the addition of 10% of mixed C&D waste caused a reduction in compressive strength of approximately 34% on laterite soil and approximately 42% on alluvial soil. There is also a reduction in compressive strength, which can be seen in laterite soil samples, and that is by 18% at 850 °C and by 17% at 900 °C. In the case of alluvial soil, the measured reduction was 28% at 850 °C and 23% at 900 °C. There is a linear decline in compressive strength as a result of the increased addition of the waste which introduced somewhat increased carbonates contents [28].

**Figure 8:**
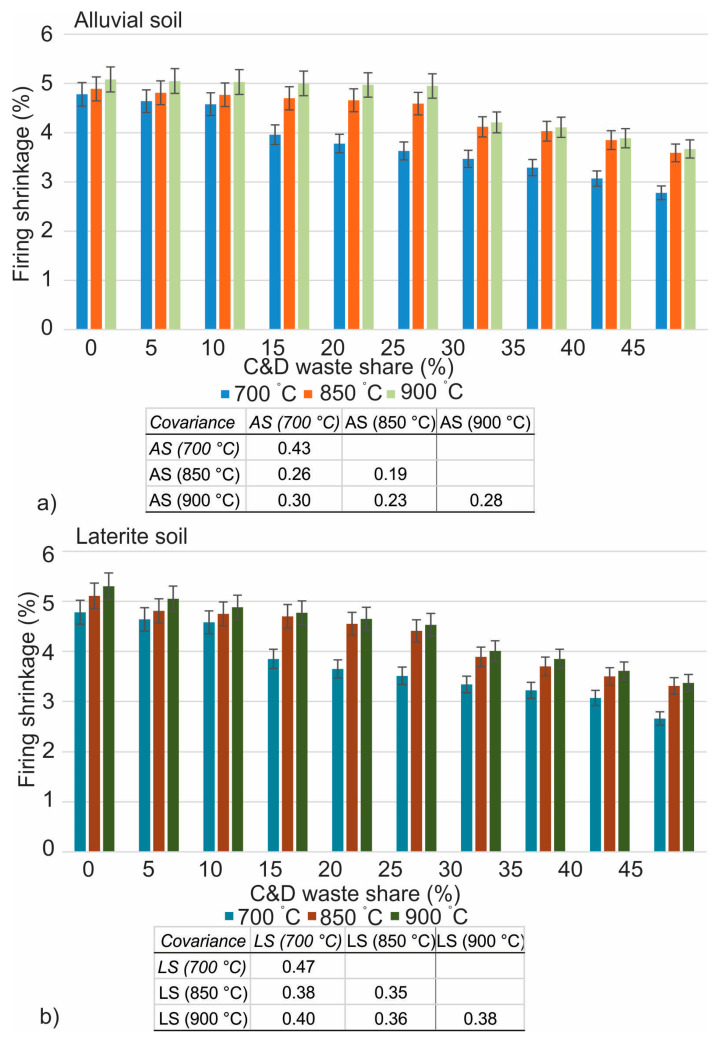
Firing linear shrinkage of mixed C&D waste in alluvial (**a**) and laterite (**b**) soils.

**Figure 9:**
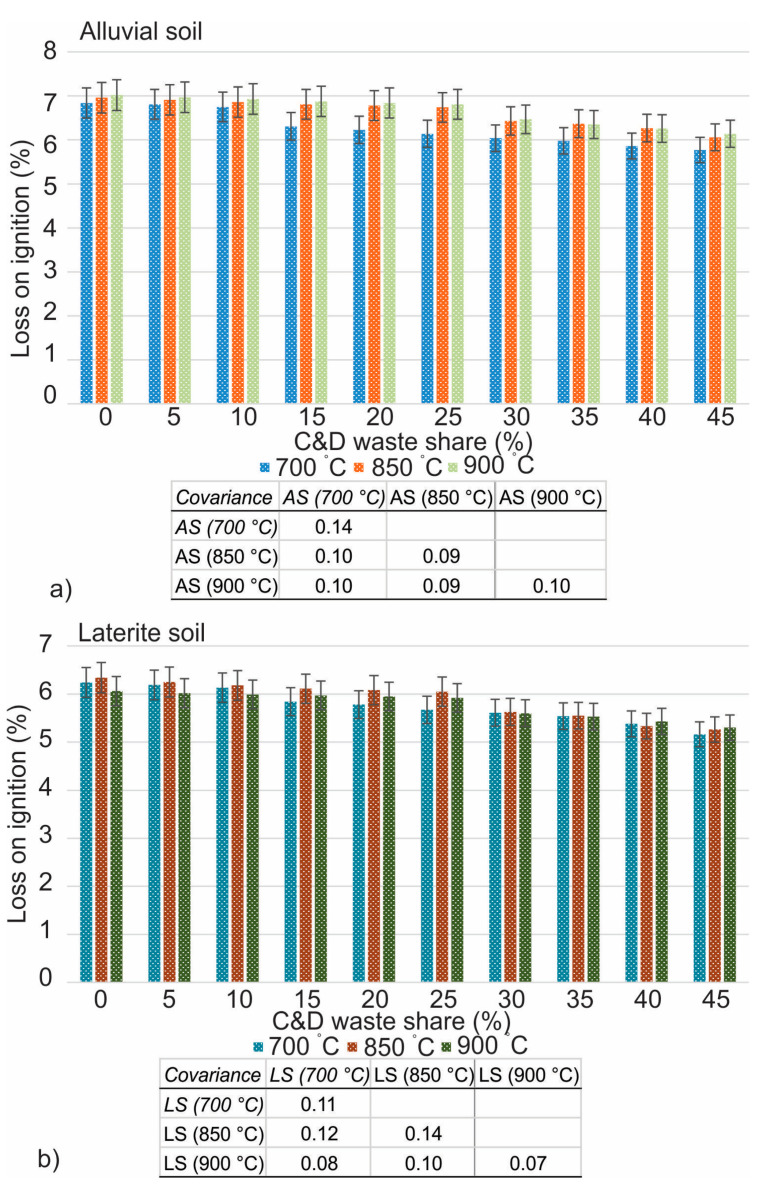
Loss on ignition of mixed C&D waste in alluvial (**a**) and laterite (**b**) soils.

**Figure 10:**
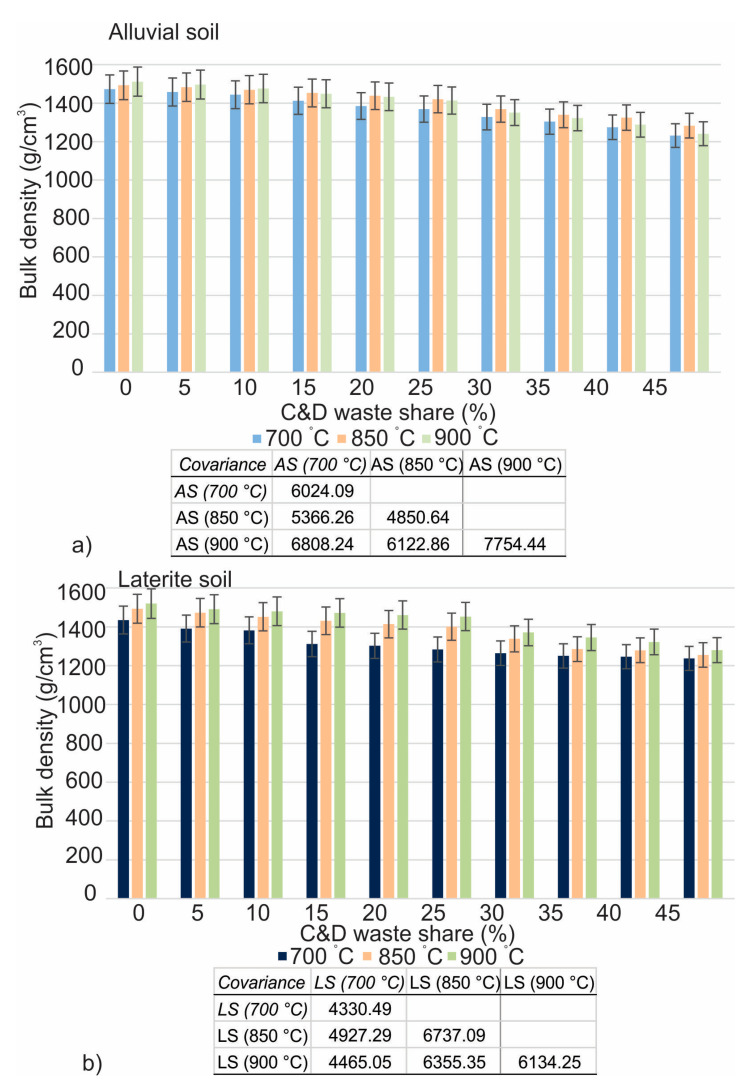
Bulk density of mixed C&D waste in alluvial (**a**) and laterite (**b**) soils.

The findings of firing linear shrinkage are provided in Figure 8, while the results of loss on ignition and bulk density are represented in Figure 9 and Figure 10, respectively. There is a subsequent decrease in density that takes place whenever mixed C&D waste is integrated into any of the soils at any one of the three temperatures. The decrease in bulk density of the fired brick specimens at a temperature of 700 °C varied from 1435 g/cm^3^ to 1382 g/cm^3^ in laterite soil and from 1473 g/cm^3^ to 1444 g/cm^3^ in alluvial soil for 0% to 10% addition of the mixed C&D waste. The bulk density, on the other hand, decreased from 15% to 45% with the addition of C&D waste in the soil, revealing 1311 g/cm^3^ to 1237 g/cm^3^ in laterite soil and 1412 g/cm^3^ to 1231 g/cm^3^ in alluvial soil at 700 °C. For 0% to 25%, assimilations of mixed C&D waste into laterite soil show the bulk density variation from 1493 g/cm^3^ to 1367 g/cm^3^, while in alluvial soil it ranges from 1493 g/cm^3^ to 1421 g/cm^3^ at 850 °C temperature.

At 850 °C, the bulk density decreases as the mix ratio rises from 30% to 45% and is demonstrated to be 1338 g/cm^3^ to 1255 g/cm^3^ in laterite soil and 1369 g/cm^3^ to 1283 g/cm^3^ in alluvial soil. For 0% to 25% inclusion of mixed C&D waste, the decrease in bulk density on brick specimens fired at 900 °C varies from 1520 g/cm^3^ to 1453 g/cm^3^ in laterite soil and from 1512 g/cm^3^ to 1414 g/cm^3^ in alluvial soil. Incorporation of the waste into laterite soil shows bulk density variations from 1406 g/cm^3^ to 1280 g/cm^3^, while in alluvial soil it ranges from 1351 g/cm^3^ to 1241 g/cm^3^. At temperatures of 700 °C, 850 °C, and 900 °C, it is possible to see a decrease in the mass loss in both types of soils when mixed C&D waste is added into brick clay. The presence of more water on the clay grain, which is then evaporated during the firing process, causes the control brick to have a greater loss on ignition, bulk density, and linear shrinkage compared to waste-added bricks. This is because control brick contains a larger amount of water and more clay minerals. In addition, the amorphous form of some volatile compounds is undergoing a transition toward the crystalline form [44,57].

Bricks that are currently going through the firing process will undergo shrinkage because of the high temperature of the firing as well as the loss of water that is mechanically and chemically bonded inside the sample. At temperatures of 700 °C from 0% to 10% addition of mixed C&D waste into both soils and at temperatures of 850 °C and 900 °C from 0% to 25%, the shrinkage that takes place is minor. However, it significantly rises in the range of 15% to 45% at 700 °C, and 30% to 45% at 850 °C and 900 °C. A shrinkage of approximately 2.91% was observed on both soils when heated to 700 °C for 0% to 10% addition, whereas 3.75% was observed for 15% to 45% addition and 0% to 10% is almost comparable to control brick (2.64%). At temperatures of 850 °C and 900 °C, the value of shrinkage is 2.67% in laterite soil and 2.73% in alluvial soil from 0% to 25% waste addition. However, the shrinkage is 3.39% in laterite soil and 3.51% in alluvial soil for 30% to 45% and 0% to 25% incorporations, which is almost equal to control brick specimens.

Upon inspection of the efflorescence, there was no evidence of flacking or cracking at all the tested temperatures with 0% to 10% waste addition at 700 °C, and from 0% to 25% addition at 850 °C and 900 °C in both soils. Cracks and bloating flaws were not seen during the sintering process at any of the three temperatures or in any of the soils. When scraped with a finger, it does not leave a mark, but when it is hit with two bricks at the same time, it emits a sound like a bell ringing.

### 3.3. Instrumental Analysis on the Optimal Fired Bricks

**Figure 11:**
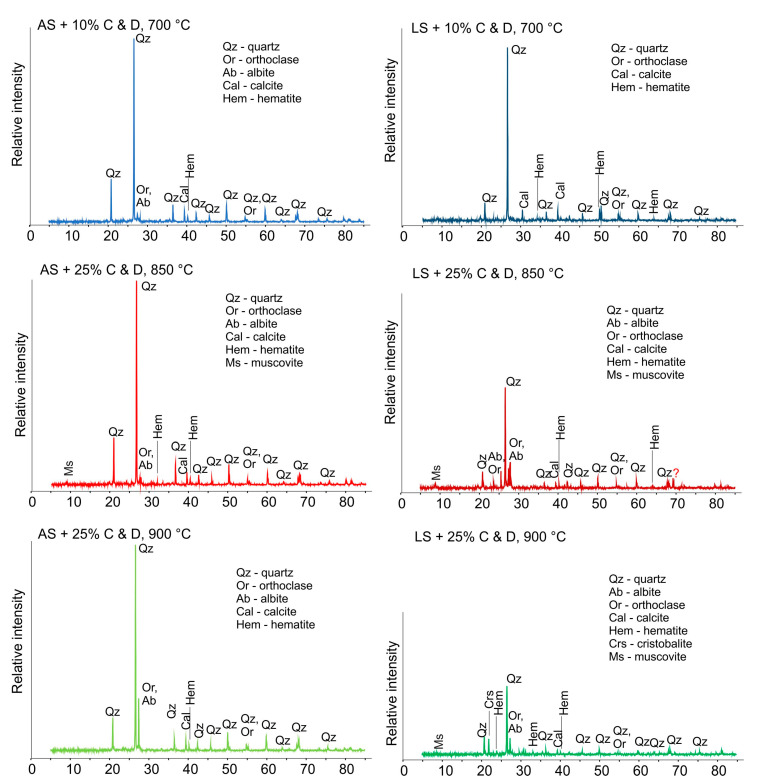
XRD analysis of the optimal mixtures.

The mineralogical composition of the fired samples found optimal (Figure 11) revealed somewhat-decreased content of quartz with increasing firing temperature. Besides, the diminishing of characteristic portlandite, ettringite, calcite and illite–mica peaks is noticed as a result of the degradation of these minerals caused by rising temperature. Additionally, orthoclase peaks became somewhat more pronounced after firing, while albite content decreased, which aligns with a previous study [31]. Laterite soil contains a lower quantity of quartz, and even more decreased with firing temperature, which induces an increased amorphous matter content and the occurrence of cristobalite [58]. A somewhat-higher quantity of hematite [59] is found in the lateritic samples, which is well aligned with the XRD analyses. Besides, minor amounts of muscovite appeared in the samples after firing at 850 °C [60].

**Figure 12:**
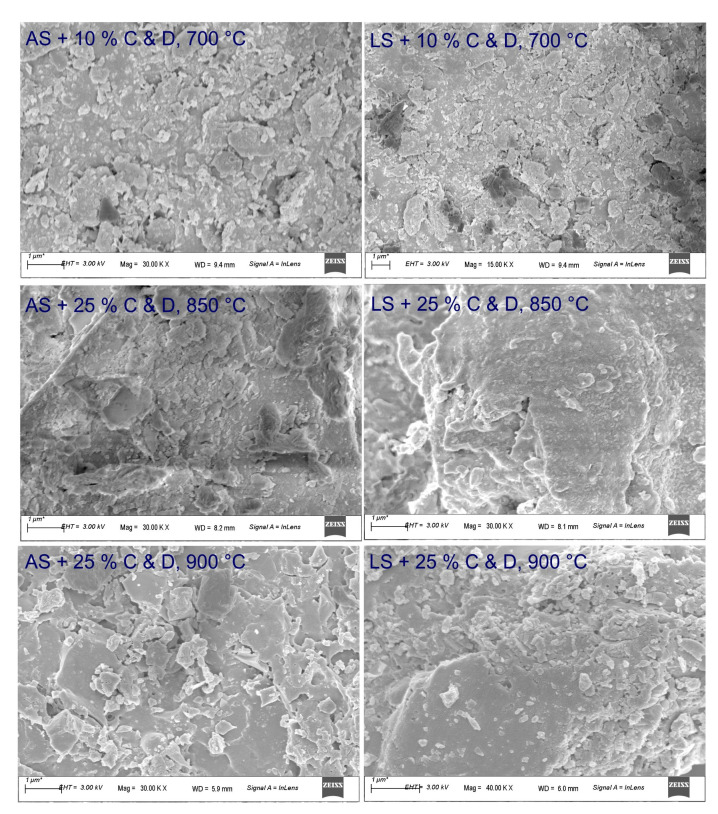
SEM images of the optimal mixtures of alluvial soil (AS) and laterite soil (LS) with construction and demolition waste (C&D) (10% C & D at 700 °C and 25% C & D at 850 °C and 900 °C).

Due to the finer distribution of grains and the higher proportion of clay minerals in the laterite soil sample, lower porosity, higher compressive strength and a higher modulus of elasticity are obtained, compared to alluvial clay [61]. The effects are reflected in the SEM images of the samples found to be optimal (Figure 12), where the rise in firing temperature strengthens the materials, and the lower porosity is seen in samples containing laterite soil. A relatively low degree of densification is seen after firing at 700 °C in both kinds of soils.

## 4. Conclusions

The primary goal of this research was to investigate construction and demolition (C&D) mixed waste in fired brick production and to reduce the use of fertile soil in brick production. Based on raw materials and fired brick characterization, mixed C&D waste can be incorporated in a quantity of 10% in the fired brick making while being fired at 700 °C, and 25% if fired at 850 °C and 900 °C in both soils. The main conclusions from the study follow:

- The addition of mixed C&D waste has shown a discernible change in the firing temperature;
- When producing burnt bricks, a larger proportion of mixed C&D waste must be added depending on the temperature. The higher the temperature, the more waste can be added. It is permissible to use up to 25% of mixed C&D waste in construction material;
- The use of mixed construction and demolition waste helps enhance the long-term conservation of natural resources and minimizes the amount of waste that is disposed of in unexpected locations;
- When producing fired bricks at temperatures between 850 °C and 900 °C, mixed construction and demolition waste makes an excellent replacement for rich agricultural soil, and may account for up to 25%.

This study gives promising results on the use of mixed C&D waste incorporated in fired brick production. The entire life-cycle assessment of these bricks will be reported in a subsequent study, but it is a way of reducing the large volume of waste dumped on unplanned sites, as well as minimizing the use of natural resources, such as construction and building material.

## Figures and Tables

**Table 1:** Literature analysis on similar waste addition on fired brick production and their key findings.

Ref.	Type of Waste	Size of Waste Particles	Characteristics of Raw Clay	Temp. (°C)	Optimum Utilization
[30]	Demolished bricks, fly ash, rice husk ash, glass cullet	≤150 μm	29.74% Al_2_O_3_ kaolinitic–illitic ball clay	800, 900 and 1000	60–80% of total waste
[31]	C&D waste	<2 mm	26.8% Al_2_O_3_	800 and 1000	30 to 70%
[32]	Ground concrete waste powder	<100 μm	11.23% Al_2_O_3_ illitic–chloritic clay	1000 and 1100	2.5–15%
[29]	Demolition floor and wall ceramic tile waste	<0.6 mm	28.64 Al_2_O_3_ kaolinitic alluvial soil 26.86 Al_2_O_3_ kaolinitic–illitic laterite soil	850 and 900	35% at 850 °C and 40% at 900 °C
[33]	Processed C&D waste and 0.1–1% of fly ash	300 μm–1.18 mm	Undefined	900	37.5% of C&D waste and 1% of fly ash
[34]	Processed C&D waste	100–250 μm	15% Al_2_O_3 c_lays containing illite mica, chlorite, kaolinite and smectite	900, 940 and 950	15%
This study	Mixed C&D waste	<0.6 mm	28.64 Al_2_O_3_ kaolinitic alluvial soil 26.86 Al_2_O_3_ kaolinitic–illitic laterite soil	700, 850 and 900	10% at 700 °C and 25% at 850 and 900 °C

**Table 2:** Chemical composition of raw materials.

Major Oxides	C&D Waste (%)	Alluvial Soil (%)	Laterite Soil (%)
SiO_2_	51.81 ± 3.48	47.07 ± 3.13	46.07 ± 3.05
Al_2_O_3_	14.3 ± 0.96	28.64 ± 1.90	26.86 ± 1.79
Fe_2_O_3_	3.81 ± 0.26	5.43 ± 0.36	10.58 ± 0.70
MnO	0.04 ± 0.00	0.56 ± 0.04	0.13 ± 0.01
MgO	2.52 ± 0.17	1.99 ± 0.13	1.83 ± 0.12
CaO	6.21 ± 0.41	1.14 ± 0.08	1.40 ± 0.09
Na_2_O	1.73 ± 0.12	0.81 ± 0.05	1.03 ± 0.07
K_2_O	1.83 ± 0.12	3.73 ± 0.25	2.77 ± 0.18
TiO_2_	0.53 ± 0.04	0.67 ± 0.04	0.50 ± 0.03
P_2_O_5_	0.09 ± 0.01	0.23 ± 0.02	0.27 ± 0.02
SO_3_	0.71 ± 0.05	0.11 ± 0.01	0.19 ± 0.01
Loss on ignition	16.42 ± 1.09	9.62 ± 0.63	8.37 ± 0.56

**Table 3:** Toxic elements in the mixed C&D waste.

Elements	Mixed C&D Waste (mg/kg)
Ac	2.12 ± 0.14
Cd	1.23 ± 0.08
Cr	1.07 ± 0.07
Cu	0.00 ± 0.00
Fe	0.00 ± 0.00
Ni	0.00 ± 0.00
Mn	1.11 ± 0.07
Pb	0.00 ± 0.00
Zn	99.6 ± 6.66

## Data Availability

The data are contained within the article. Additional data are available on request.

## References

[1] Jin R., Li B., Zhou T., Wanatowski D., Piroozfar P. (2017). An empirical study of perceptions towards construction and demolition waste recycling and reuse in China. Resour. Conserv. Recycl..

[2] Akhtar A., Sarmah A.K. (2018). Construction and demolition waste generation and properties of recycled aggregate concrete: A global perspective. J. Clean. Prod..

[3] Wu H., Wang J., Duan H., Ouyang L., Huang W., Zuo J. (2016). An innovative approach to managing demolition waste via GIS (geographic information system): A case study in Shenzhen city, China. J. Clean. Prod..

[4] Islam R., Nazifa T.H., Yuniarto A., Shanawaz Uddin A.S.M., Salmiati S., Shahid S. (2019). An empirical study of construction and demolition waste generation and implication of recycling. Waste Manag..

[5] Turkyilmaz A., Guney M., Karaca F., Bagdatkyzy Z., Sandybayeva A., Sirenova G. (2019). A comprehensive construction and demolition waste management model using PESTEL and 3R for construction companies operating in Central Asia. Sustainability.

[6] Roychowdhury A., Somvanshi A., Verma A. (2020). Another Brick off The Wall: Improving Construction and Demolition Waste Management in Indian Cities. Centre for Science and Environment, New Delhi. https://www.cseindia.org/another-brick-off-the-wall-10325.

[7] Gálvez-Martos J.-L., Styles D., Schoenberger H., Zeschmar-Lahl B. (2018). Construction and demolition waste best management practice in Europe. Resour. Conserv. Recycl..

[8] Torres A., Brandt J., Lear K., Liu J. (2017). A looming tragedy of the sand commons. Science.

[9] Mineral Commodity Summaries 2022 U.S. Geological Survey. https://pubs.usgs.gov/periodicals/mcs2022/mcs2022.pdf.

[10] Shrivastava S., Chini A. (2009). Construction Materials and C&D Waste in India.

[11] D’Souza R. (2019). Housing poverty in urban India: The failures of past and current strategies and the need for a new blueprint. ORF Obs. Res. Found. Occas. Pap. No.

[12] Maithel S. (2013). Evaluating Energy Conservation Potential of Brick Production in India. A Report Prepared for the SAARC Energy Centre, Islamabad. https://www.saarcenergy.org/wp-content/uploads/2022/06/2012-Final-Report-Evaluating-Energy-Conservation-in-Brick-Production-in-India.pdf.

[13] Guney Y., Sari Y.D., Yalcin M., Tuncan A., Donmez S. (2010). Re-usage of waste foundry sand in high-strength concrete. Waste Manag..

[14] Krishna Sastry K.V.S.G., Ravi Theja A., Reddy T.C.S. (2018). Effect of used foundry sand and mineral admixtures on strength properties of concrete. Arch. Civ. Eng..

[15] Siddique R., Dhanoa G. Design & development of concrete using waste foundry sand as partial replacement of fine aggregate. Proceedings of the Thirteenth East Asia-Pacific Conference on Structural Engineering and Construction (EASEC-13).

[16] Nguyen T.S., Thai M.Q., Ho L.S. (2021). Properties of fine-grained concrete containing fly ash and bottom ash. Mag. Civ. Eng..

[17] Ovbeniyekede O.S., Adenan D.S.Q., Ahmad M., Kamaruddin K. (2018). Water absorption and compressive strength of self-compacting concrete incorporating fly ash and quarry dust. IJSRP.

[18] Feng J., Sun J., Yan P. (2018). The influence of ground fly ash on cement hydration and mechanical property of mortar. Adv. Civ. Eng..

[19] Rai A.K., Paul B., Singh G. (2010). A study on back fill properties and use of fly ash for highway embankments. J. Adv. Lab. Res. Biol..

[20] Athanasopoulou A., Kollaros G. (2015). Fly ash exploited in pavement layers in environmentally friendly ways. Toxicol. Environ. Chem..

[21] Zhai H., Tang Y., Chen S., Chen H., Cheng B., Cai X., Wei Y. (2021). Experimental research on durability of fly ash pavement concrete and mix proportion, optimization. Adv. Mater. Sci. Eng..

[22] Mirković K., Tošić N., Mladenović G. (2019). Effect of different types of fly ash on properties of asphalt mixtures. Adv. Civ. Eng..

[23] Vasić M.V., Pezo L., Gupta V., Chaudhary S., Radojević Z. (2021). An artificial neural network-based prediction model for utilization of coal ash in production of fired clay bricks: A review. Sci. Sinter..

[24] Rao A., Jha K.N., Misra S. (2007). Use of aggregates from recycled construction and demolition waste in concrete. Resour. Conserv. Recycl..

[25] Shahidan S., Azmi M.A.M., Kupusamy K., Zuki S.S.M., Ali N. (2017). Utilizing construction and demolition (C&D) waste as recycled aggregates (RA) in concrete. Procedia Eng..

[26] Ghorbani B., Arulrajah A., Narsilio G., Horpibulsuk S., Bo M.W. (2021). Shakedown analysis of PET blends with demolition waste as pavement base/subbase materials using experimental and neural network methods. Transp. Geotech..

[27] Migunthanna J., Rajeev P., Sanjayan J. (2021). Investigation of waste clay brick as partial replacement of geopolymer binders for rigid pavement application. Constr. Build. Mater..

[28] Marrocchino E., Zanelli C., Guarini G., Dondi M. (2021). Recycling mining and construction wastes as temper in clay bricks. Appl. Clay Sci..

[29] Dubale M., Goel G., Kalamdhad A., Singh L.B. (2022). An investigation of demolished floor and wall ceramic tile waste utilization in fired brick production. Environ. Technol. Innov..

[30] Hossain S.S., Mathur L., Majhi M.R., Roy P.K. (2019). Manufacturing of green building brick: Recycling of waste for construction purpose. J. Mater. Cycles Waste Manag..

[31] Simões dos Reis G., Cazacliu B.G., Cothenet A., Poullain P., Wilhelm M., Sampaio C.H., Lima A.C., Ambros W., Torrenti J.-M. (2020). Fabrication, microstructure, and properties of fired clay bricks using construction and demolition waste sludge as the main additive. J. Clean. Prod..

[32] Gencel O., Erdugmus E., Sutcu M., Oren O.H. (2020). Effects of concrete waste on characteristics of structural fired clay bricks. Constr. Build. Mater..

[33] Harikumar M., Mohamed F., Mohammed A., Ashraf I., Shahansha M., Anand A.G. (2022). Clay bricks using building debris. Mater. Today: Proc..

[34] Zanelli C., Marrocchino E., Guarini G., Toffano A., Vaccaro C., Dondi M. (2021). Recycling Construction and Demolition Residues in Clay Bricks. Appl. Sci..

[35] (2015). 17 Sustainable Development Goals. 17 Partnerships. Division for Sustainable Development, United Nations Department of Economic and Social Affairs. https://sustainabledevelopment.un.org/content/documents/211617%20Goals%2017%20Partnerships.pdf.

[36] Bolden J., Abu-Lebdeh T., Fini E. (2013). Utilization of recycled and waste materials in various construction applications. Am. J. Environ. Sci..

[37] Goel G., Kalamdhad A.S. (2017). An investigation on use of paper mill sludge in brick manufacturing. Constr. Build. Mater..

[38] (1992). Method 1311—Toxicity Characteristic Leaching Procedure, Environmental Protection Agency (EPA). https://www.epa.gov/sites/default/files/2015-12/documents/1311.pdf.

[39] Bhavikatti S.S. (2010). Basic Civil Engineering. New Age International Publishers. https://basicengn.files.wordpress.com/2019/09/basic-civil-engineering-materials.pdf.

[40] Pacheco-Torgal F., Lourenço P.B., Labrincha J.A., Kumar S., Chindaprasirt P. (2015). Eco-Efficient Masonry Bricks and Blocks: Design, Properties and Durability.

[41] Pfeifer G., Ramcke R., Achtzicker J., Zilch K. (2001). Masonry Construction Manual.

[42] (2015). Water Absorption, Apparent Specific Gravity, and Bulk Density of Burned Refractory Brick and Shapes by Boiling Water.

[43] Goel G., Kalamdhad A.S. (2018). A practical proposal for utilisation of water hyacinth: Recycling in fired bricks. J. Clean. Prod..

[44] (1992). Methods of Tests of Burnt Clay Building Bricks: Part 1 Determination of Compressive Strength, Part 2 Determination of Water Absorption, Part 3 Determination of Efflorescence, Part 4: Determination of Warpage. 13.

[45] Johari I., Said S., Jaya R.P., Bakar B.H.A., Ahmad Z.A. (2011). Chemical and physical properties of fired-clay brick at different type of rice husk ash. Environ. Sci. Eng..

[46] Vasić M.V., Terzić A., Radovanović Ž., Radojević Z., Warr L.N. (2022). Alkali-activated geopolymerization of a low illitic raw clay and waste brick mixture. An alternative to traditional ceramics. Appl. Clay Sci..

[47] Iftikhar S., Rashid K., Ul Haq E., Zafar I., Alqahtani F.K., Iqbal Khan M. (2020). Synthesis and characterization of sustainable geopolymer green clay bricks: An alternative to burnt clay brick. Constr. Build. Mater..

[48] Samadi M., Huseien G.F., Mohammadhosseini H., Lee H.S., Abdul Shukor Lim N.H., Tahir M.M., Alyousef R. (2020). Waste ceramic as low cost and eco-friendly materials in the production of sustainable mortars. J. Clean. Prod..

[49] Robayo-Salazar W., Valencia-Saavedra W., de Gutiérrez R.M. (2020). Construction and demolition waste (CDW) recycling—As both binder and aggregates—In alkali-activated materials: A novel re-use concept. Sustainability.

[50] Siddique S., Shrivastava S., Chaudhary S. (2018). Influence of ceramic waste as fine aggregate in concrete: Pozzolanic, XRD, FT-IR, and NMR investigations. Mater. Civ. Eng..

[51] Nigay P.-M., Nzihou A., Cutard T. (2018). The impact of the particle size distribution of organic additives on the microstructure of a clay ceramic and its thermal and mechanical properties. J. Mater. Civ. Eng..

[52] Vedalakshmi R., Sundara Raj A., Srinivasan S., Ganesh Babu K. (2003). Quantification of hydrated cement products of blended ce-ments in low and medium strength concrete using TG and DTA technique. Thermochim. Acta.

[53] Anjum F., Yasin Naz M., Ghaffar A., Shukrullah S. (2021). Study of microstructural, physical, thermal, and mechanical properties of organic waste–incorporated fired clay bricks in the framework of energy conservation. J. Mater. Civ. Eng..

[54] ASTM (2012). ASTM C62—Standard Specification for Building Brick (Solid Masonry Units Made from Clay or Shale).

[55] (1992). Common Burnt Clay Building Bricks -Specification, Bureau of Indian Standard (BIS).

[56] Arsenović M., Pezo L., Stanković S., Radojević Z. (2013). Sensitivity analysis of mathematical models for final product properties: Link to DTG curve. Ceram. Int..

[57] Debnath A., Salma M.M., Islam M.S., Mostafa M.G., Samad A., Akhtar U.S. (2019). Effect of processed rice husk ash on the production of conventional bricks. Int. J. Sci. Eng. Res..

[58] Yannick B.I., Ludovic W.A.S., Francois N., Véronique K.K., Nathalie F. (2019). Mineralogical Transformation and Microstructure of the Alluvials Clays. Sci. Sinter..

[59] Chen Y., Zhang Y., Chen T., Liu T., Huang J. (2013). Preparation and characterization of red porcelain tiles with hematite tailings. Constr. Build. Mat..

[60] Vieira C.M.F., Monteiro S.N. (2019). Firing Behavior of the Clay Fraction of a Natural Kaolinitic Clay: Are They Different?. Mat. Res..

[61] Goel G., Vasić M.V., Katiyar N.K., Kirthika S.K., Pezo M., Dinakar P. (2021). Potential pathway for recycling of the paper mill sludge compost for brick making. Constr. Build. Mat..

